# CD4^+^CD25^+^CD127^−^ and CD4^+^CD25^+^Foxp3^+^ Regulatory T Cell Subsets in Mediating Autoimmune Reactivity in Systemic Lupus Erythematosus Patients

**DOI:** 10.1007/s00005-016-0399-5

**Published:** 2016-05-07

**Authors:** Marcelina Żabińska, Magdalena Krajewska, Katarzyna Kościelska-Kasprzak, Katarzyna Jakuszko, Dorota Bartoszek, Marta Myszka, Marian Klinger

**Affiliations:** Department and Clinic of Nephrology and Transplantation Medicine, Faculty of Postgraduate Medical Training, Wroclaw Medical University, Wrocław, Poland

**Keywords:** Systemic lupus erythematosus, Lupus nephritis, Regulatory cells, Flow cytometry

## Abstract

The available clinical as well as experimental studies implicate participation of T regulatory (Treg) subsets in the pathogenesis and course of systemic lupus erythematosus (SLE). Introduction of the CD4^+^CD25^+^CD127^−^ and CD4^+^CD25^+^Foxp3^+^ regulatory subpopulations analysis into immunological processes assessment and disease activation prognosis in patients with lupus nephritis (LN) may improve monitoring of disease activity and enable an early, and thus more effective, therapeutic treatment. The main goal of the study was to investigate whether the quantitative changes of Treg subpopulations are related to the clinical status of patients with LN. Fifty-four adult SLE patients divided into two groups according to their SLEDAI and renal SLEDAI scores were enrolled into the study. Subpopulations of CD4^+^CD25^+^CD127^−^ and CD4^+^CD25^+^Foxp3^+^ phenotypes were determined by flow cytometry. The control group had higher absolute number of CD4^+^CD25^+^Foxp3^+^ cells compared with the study group (*p* < 0.001). Also, significant inverse correlation in the absolute number of CD4^+^CD25^+^Foxp3^+^ cells and SLEDAI score was observed. There were significant differences in the percentage and absolute number of CD4^+^CD25^+^Foxp3^+^ lymphocytes between active and non-active LN groups. The study group had statistically lower values of CD4^+^CD25^+^CD127^−^ cells, both in the percentage (*p* < 0.001) as well as their absolute number (*p* = 0.014) compared to the control group. There were also statistically significant positive correlations between the absolute number of CD4^+^CD25^+^CD127^−^ and CD4^+^CD25^+^Foxp3^+^ Tregs. In conclusion: (1) reduction in the number of regulatory CD4^+^CD25^+^Foxp3^+^ cells is a promising indicator of the activity of SLE, particularly of renal involvement; (2) determination of the number of regulatory cells using the CD4^+^CD25^+^CD127^−^ phenotype is unreliable in patients with SLE.

## Introduction

In the mid-1990, a subpopulation of T cells expressing the α chain of interleukin (IL)-2 receptor (CD25 molecule) has been shown to be able to prevent the occurrence of systemic autoimmune diseases in athymic mice. These cells have been called T regulatory cells (Tregs) and considered a promising means of immunotherapy for many clinical diseases (Sakaguchi et al. 1995). However, the upregulation of CD25 molecule also occurs on activated T cells, and the boundary between the populations with low and high CD25 expression is not strictly defined. The search for a specific marker identifying T cell population with suppressor properties was performed (Sakaguchi et al. 2010; Yamazaki et al. 2003), and nuclear transcription factor Foxp3 was proved to comply with this requirements (Fontenot et al. 2003; Hori et al. 2003). Unfortunately, intracellular Foxp3 staining cannot be used to isolate the subpopulation of Tregs to carry out functional tests, because it requires the fixation and permeabilization of the cells. It has been shown that simultaneous high expression of the CD25 and low expression of CD127 molecules correspond with intracellular factor Foxp3 expression, which allows to identify T cell populations with suppressor activity (Liu et al. 2006; Miyara and Sakaguchi 2011). The correlation between CD4^+^CD25^+^CD127^low^ and CD4^+^CD25^+^Foxp3^+^ cells has been confirmed in healthy individuals (Seddiki et al. 2006), but as in the case of CD25 molecule, CD127 expression is significantly reduced after stimulation, which makes it difficult to be used as an identifier of regulatory cells (Aerts et al. 2008).

Treg cells are a specialized T cell subpopulation capable of controlling and limiting the harmful immune response. Treg cells also prevent the immune response against self-antigens and have a strong suppressive activity under inflammatory conditions (Kaczorowski and Jutel 2013). Regulatory T cells subpopulation is believed to play a key role in maintaining peripheral tolerance through the mechanism of controlling circulating autoreactive T cells that have not been removed in the thymus (Gerli et al. 2009).

It has been demonstrated in a mouse model of chronic graft-versus-host disease (GVHD) that regulatory cells have the ability to control reactivity of donor CD8^+^ T cells to recipient antigens. Results suggest that donor’s Treg cells may be necessary to maintain the anergy of CD8^+^ T lymphocytes and inhibit the development of acute GVHD (Kim et al. 2006).

Treg cells can also be used therapeutically in organ transplantation to suppress T cell response to alloantigens (Lin et al. 2013).

Immune disturbances in systemic lupus erythematosus (SLE) may be associated with abnormal homeostasis or defective function of regulatory cells, which has been proved in the study carried on mice susceptible to lupus with genetically modified regulatory CD4^+^CD25^+^ cell depletion caused by thymectomy. These mice showed the expansion of autoreactive T cells and increased the production of autoantibodies. Treg CD4^+^CD25^+^ cells from normal syngeneic mice supplied to athymic animals inhibited progression of autoimmune disease (Bagavant and Tung 2005).

So far, many studies addressing the size and activity of the CD4^+^CD25^+/high^ subpopulation in patients with SLE have been performed. It has been shown that the number of CD4^+^CD25^+^ is reduced in children with SLE and negatively correlates with disease activity measured by the SLEDAI scale and anti-dsDNA serum levels. Surprisingly, higher expression levels of Foxp3 mRNA molecules in the population of CD4^+^ cells has been demonstrated in the active SLE in comparison with the control group and patients with inactive SLE (Lee et al. 2008).

It has been also observed that depletion of CD4^+^CD25^high^ in lupus patients was associated with exacerbation of the disease (Miyara et al. 2005).

Most researchers indicate a reduced number of circulating CD4^+^CD25^high^/CD4^+^CD25^+^Foxp3^+^ during the activation of the disease (Barath et al. 2007a, 2007b; Barreto et al. 2009; Bonelli et al. 2008; Cai et al. 2012; Crispin et al. 2003; Habibagahi et al. 2011; Henriques et al. 2010; Lee et al. 2006, 2008; Lyssuk et al. 2007; Ma et al. 2013; Mellor-Pita et al. 2006; Miyara et al. 2005; Suen et al. 2009; Xing et al. 2012; Yang et al. 2009). The decrease negatively correlates with disease activity and/or levels of anti-dsDNA antibodies in the patients’ sera (Bonelli et al. 2008; Lee et al. 2006; Ma et al. 2013; Mellor-Pita et al. 2006; Miyara et al. 2005; Yang et al. 2009).

Others have reported unchanged number of circulating regulatory T CD4^+^ cells which express the CD25 molecules and/or Foxp3 (Alvarado-Sanchez et al. 2006; Vargas-Rojas et al. 2008; Venigalla et al. 2008; Yates et al. 2008; Zhang et al. 2008), or on the contrary—higher number of these cells in patients with SLE (Azab et al. 2008; Lin et al. 2007; Suarez et al. 2006; Yan et al. 2008) compared to the control group.

These conflicting results may be due to the unclear definition of a Treg cell phenotype. Because of the unique pattern of CD25 expression in human activated CD4^+^ T cells and Treg cells, it is difficult to determine the boundary between CD25^high^ and CD25^low^ cells on the fluorescence plots of each subpopulation. Simultaneous analysis of CD25 and Foxp3 expression on CD4^+^ T cells simplifies distinguishing the subsets of T lymphocytes. In case only the CD25 molecule is used as a marker for regulatory cells, CD25^high^ cells can be contaminated by CD25^low^ effector T cells. While too strict gating of CD4^+^ T cells with high expression of CD25 molecule may lead to false reduced number of regulatory cells in human peripheral blood mononuclear cells (Suen and Chiang 2012).

The main goal of the study was to investigate whether the quantitative changes of Treg subpopulations are related to clinical status of patients with lupus nephritis (LN).

## Materials and Methods

### Patients

Fifty-four adult SLE patients (96.3 % female, mean age 36.5 ± 13.7) in the various phases of disease activity were enrolled into the study. Disease activity at the time of evaluation was scored according to the Systemic Lupus Erythematosus Disease Activity Index (SLEDAI) (Bombardier et al. 1992) and renal SLEDAI (refers to the total of all renal components used to calculate the SLEDAI). Patients were divided into two groups according to their SLEDAI score, and there were 15 patients in inactive (SLEDAI ≤5) and 39 in active (SLEDAI >5) phase of disease (Griffiths et al. 2005). When disease activity was measured by the renal SLEDAI (rSLEDAI) scale, the groups’ sizes were 14 (rSLEDAI <4) and 40 (rSLEDAI 4–16) patients, respectively. The demographic characteristics and clinical data of the study group have been presented in Table 1. Also, 19 sex- and age-matched healthy volunteers (89.5 % female, mean age 38.3 ± 14.1) served as the control group.

**Table 1:** Characteristics of the study group in terms of age, gender and disease activity measured by SLEDAI and rSLEDAI scales

	SLEDAI		rSLEDAI	
SLEDAI/rSLEDAI score	≤5	>5	0	4–16
Group size	15	39	14	40
Mean SLEDAI/rSLEDAI	3.2	13	0	7.7
Median SLEDAI/rSLEDAI	4	12	0	8
Min–max SLEDAI/rSLEDAI	0–5	6–28	0–0	4–16
Mean age	32.7 ± 9.1	37.9 ± 14.9	34.7 ± 10.2	37.1 ± 14.8
Median age	32	33	33	33
Sex	♀: 13 (86.7 %)	♀: 39 (100 %)	♀: 14 (100 %)	♀: 38 (95 %)
	♂: 2 (13.3 %)	♂: 0 (0 %)	♂: 0 (0 %)	♂: 2 (5 %)

Patients were treated at the Department of Nephrology and Transplantation Medicine, Medical University of Wroclaw in accordance with the current guidelines for lupus nephropathy.

Patients with newly diagnosed renal disease in the course of SLE used steroids infusion, with the conversion to oral steroids at tapering doses or were administered only oral steroids. Furthermore, the immunosuppressive therapy included cyclophosphamide, azathioprine, cyclosporin A, mycophenolate mofetil and chloroquine. To maintain remission, patients used oral steroids or oral steroids combined with mycophenolate mofetil or azathioprine. Exclusion criteria of the study were: presence of an active malignancy and inflammatory processes. The study was approved by the Medical University of Wroclaw Bioethics Committee.

### Treg Cell Staining

The number and percentage of regulatory T CD4^+^ cells was determined in two different ways. A subpopulation of CD4^+^CD25^+^CD127^−^ cells was identified by extracellular staining with anti-CD3, anti-CD4, anti-CD25 and anti-CD127 antibodies. The number of CD4^+^CD25^+^Foxp3^+^ subpopulation was determined by intracellular staining using anti-CD3, anti-CD4, anti-CD25 and anti-Foxp3 antibodies. The measurements were accompanied by BD multitest TBNK (Becton-Dickinson, San Jose, CA, USA) for absolute cell count determination. The analysis was performed with FACS Calibur flow cytometer using CellQuest software.

### Determination of CD4^+^CD25^+^Foxp3^+^ Regulatory Cells

**Fig. 1:**
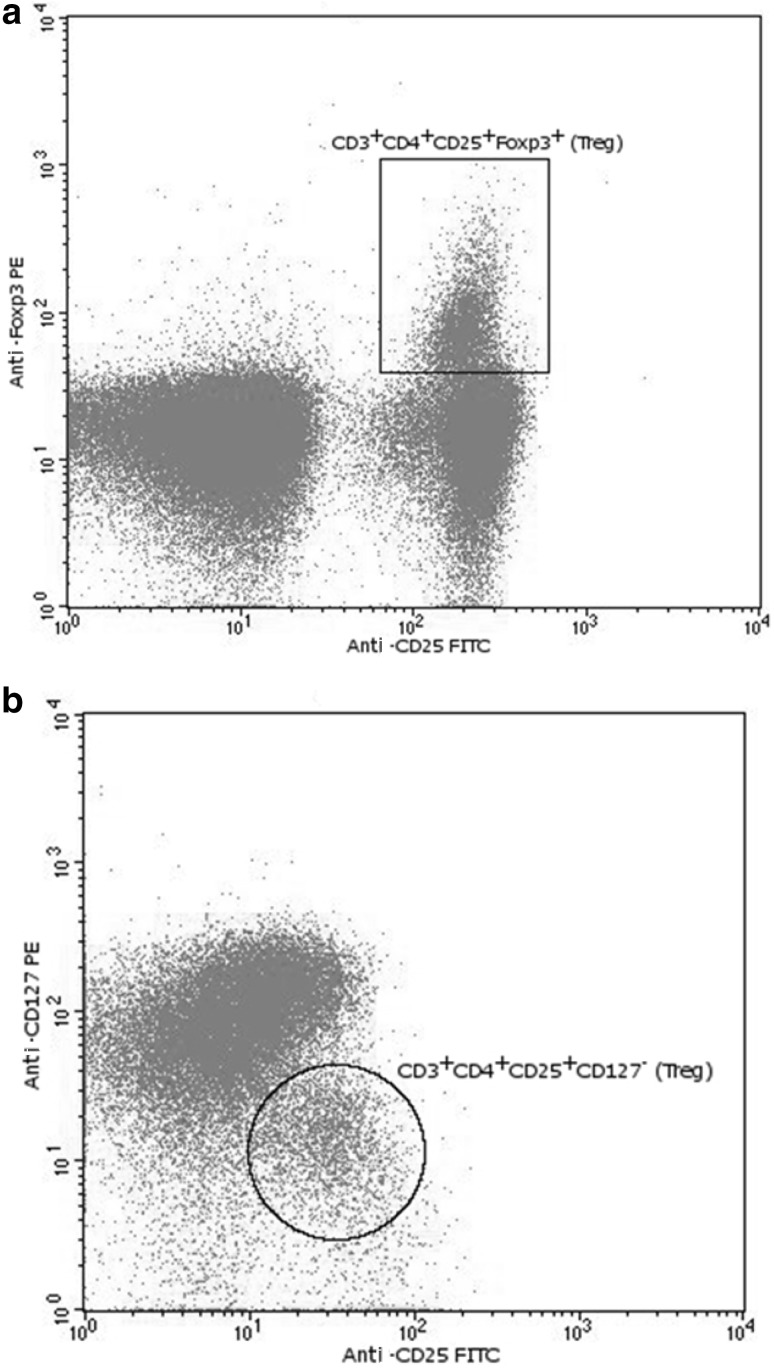
**a** Anti-Foxp3PE and anti-CD25FITC fluorescence plot and CD3^+^CD4^+^CD25^+^Foxp3^+^ Treg gating. **b** Anti-CD127PE and anti-CD25FITC fluorescence plot and CD3^+^CD4^+^CD25^+^CD127^−^ Treg gating

Heparinized blood (300 µl) was stained with 20 µl of the following antibodies: anti-CD4PerCP, anti-CD3APC and anti-CD25FITC. All were purchased from Becton-Dickinson (BD, San Jose, CA, USA). After 30 min of incubation at 4 °C in the dark, the red blood cells were lysed with BD FACS Lysing Solution (Becton-Dickinson). The cells were washed with phosphate-buffered saline (PBS) 2 % fetal bovine serum (FBS) and permeabilized with the Fixation/Permeabilization Concentrate (eBioscience, San Diego, CA, USA) in Fixation/Permeabilization Diluent (eBioscience) for 30 min at 4 °C in the dark. After two washing steps in Permeabilization Buffer (eBioscience), the cell pellet were stained with 5 µl of an anti-Human Foxp3PE clone 236A/E7 (eBioscience) for 30 min at 4 °C in the dark. The samples were then washed twice in Permeabilization Buffer (eBioscience) and flow cytometry analyzed (Fig. 1a).

For each sample, the absolute cell number of CD3^+^CD4^+^, CD4^+^CD25^+^Foxp3^+^ and their percentage in the population of T cells was determined in relation to the number of CD3^+^ lymphocytes.

### Determination of CD4^+^CD25^+^CD127^−^ Regulatory Cells

Heparinized blood (300 µl) was stained with 20 µl of the following antibodies: anti-CD4PerCP, anti-CD3APC, anti-CD25FITC and 5 µl of anti-CD127PE. All were purchased from Becton-Dickinson (BD, San Jose, CA, USA). After 30 min of incubation at 4 °C in the dark, the red blood cells were lysed with BD FACS Lysing Solution (Becton-Dickinson). The cells were washed twice with PBS 2 % FBS and flow cytometry analyzed (Fig. 1b). For each sample, the absolute cell count of the population of CD4^+^CD25^+^CD127^−^ and their percentage in the population of T cells was determined in relation to the number of lymphocytes CD3^+^.

### Statistics

The experimental and clinical data were combined and statistically analyzed using STATISTICA 10 software. The results of statistical analysis are presented with interquartile range. Correlation analysis was performed using the Spearman procedure. The Mann–Whitney *U* test (for independent samples) was applied and differences with *p* less than 0.05 were considered statistically significant.

## Results

**Fig. 2:**
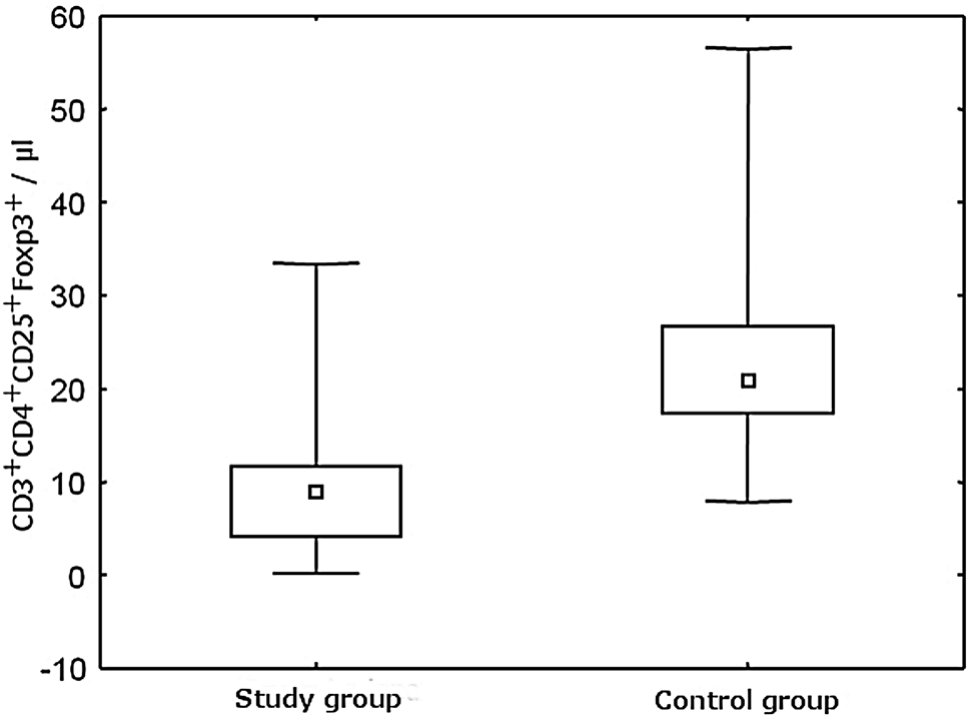
Comparison of the absolute number of CD3^+^CD4^+^CD25^+^FoxP3^+^ regulatory cells in the study and the control group

The parameters measured regarding regulatory CD4^+^CD25^+^Foxp3^+^ cells were the percentage of the total TCD4^+^ lymphocytes population and the absolute number of regulatory CD4^+^CD25^+^Foxp3^+^ in whole blood (values given per microliter). Statistically significant differences in both the percentage of regulatory cells and their absolute numbers between the study group and the control group have been demonstrated (Table 2). The patients with SLE presented significantly lower absolute count and percentage of CD4^+^CD25^+^Foxp3^+^ cells compared to healthy control (*p* < 0.001) (Fig. 2). SLE patients had three times lower percentage and more than two times lower absolute count of these cells compared to the healthy control.

**Table 2:** The number of CD4^+^CD25^+^Foxp3^+^ Treg cells in the study and the control groups

	Study group	Control group	*p*
Group size	54	19	
Percentage			
Median	1.10	3.36	**<0.001**
IQ range	0.85–2.56	2.96–3.66	
Absolute number			
Median	8.94	20.90	**<0.001**
IQ range	4.05–11.80	17.26–26.72	

Statistically significant values are marked in bold

**Fig. 3:**
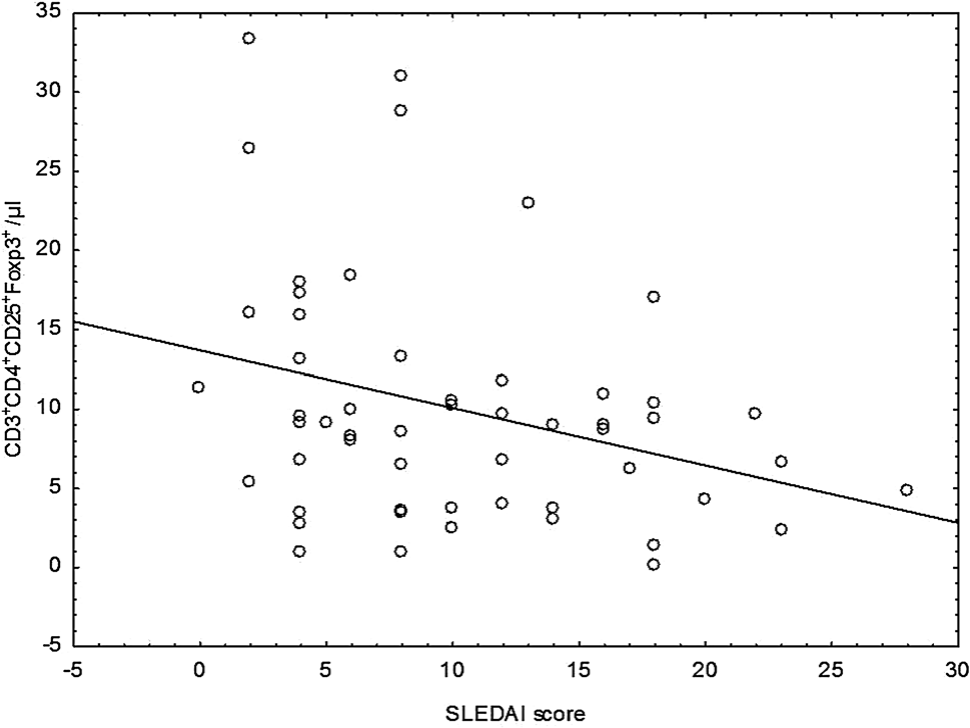
Correlation between the absolute number of CD3^+^CD4^+^CD25^+^Foxp3^+^ regulatory cells and disease activity index SLEDAI

Additionally, variability in the number of regulatory CD4^+^CD25^+^Foxp3^+^ lymphocytes depending on the activity of the disease measured by SLEDAI scale has been observed. Groups with different disease activity did not differ from each other significantly, although the proportion of observed values and the absolute number of CD4^+^CD25^+^Foxp3^+^ regulatory cells were higher in the group with low disease activity. Moreover, significant inverse correlation between decreasing absolute number of CD4^+^CD25^+^Foxp3^+^ and disease activity measured by SLEDAI scale (rs = −0.294) has been found (Fig. 3).

Statistical analysis showed a significant difference in the percentage of regulatory CD4^+^CD25^+^Foxp3^+^ cells between the low disease activity group and the control group (*p* = 0.008) and between the high disease activity group and the controls (*p* < 0.001). In both cases, there was a significantly lower number of Tregs in the SLE groups. The patient groups also differed from the control group in the absolute number of CD4^+^CD25^+^Foxp3^+^ subpopulation. In the group with low disease activity, significantly lower absolute count of CD4^+^CD25^+^Foxp3^+^ cells was observed as compared to the control group (*p* = 0.001). In the study group with high disease activity, there were also significantly lower absolute values of the regulatory cells compared with the control group (*p* < 0.001).

Quantities of regulatory CD4^+^CD25^+^Foxp3^+^ subset depending on the disease activity measured by rSLEDAI have been presented in Table 3.

**Table 3:** The number of CD4^+^CD25^+^Foxp3^+^ Treg cells in the study group depending on the severity of the disease determined by the rSLEDAI scale

	rSLEDAI = 0	rSLEDAI 4–16	*p*
Group size	14	40	
Percentage			
Median	2.31	1.02	**0.033**
IQ range	1.09–2.82	0.70–1.50	
Absolute number			
Median	12.20	8.37	**0.045**
IQ range	6.78–17.33	3.77–10.32	

Statistically significant values are marked in bold

There were significant differences in the percentage of regulatory cells in groups according to rSLEDAI. In the group with inactive nephritis, significantly higher percentage and absolute count of CD4^+^CD25^+^Foxp3^+^ regulatory cells have been observed compared to the group with active LN. In patients with high disease activity, almost 30 % less regulatory cells has been found compared to the group in which rSLEDAI equals 0. Both groups were also significantly different from the healthy group, in which higher percentage and absolute number of CD4^+^CD25^+^Foxp3^+^ regulatory cells have been demonstrated compared with those with inactive LN (*p* = 0.013 and *p* = 0.002, respectively) and the group with active nephritis (*p* < 0.001 in both cases).

The parameters that were studied according to the CD4^+^CD25^+^CD127^−^ population of regulatory cells were percentage of these cells related to the total population of TCD4^+^ lymphocytes and the absolute number of regulatory CD4^+^CD25^+^CD127^−^ cells in whole blood (values given per microliter).

In the study group, there were statistically lower values of CD4^+^CD25^+^CD127^−^ regulatory cells both in the percentage (*p* < 0.001) and their absolute number (*p* = 0.014) compared to the control group (Table 4).

**Table 4:** The number of CD4^+^CD25^+^CD127^−^ Treg cells in the study and the control groups

	Study group	Control group	*p*
Group size	54	19	
Percentage			
Median	5.79	8.11	**<0.001**
IQ range	4.72–7.59	7.25–9.14	
Absolute number			
Median	39.96	47.53	**0.014**
IQ range	22.02–54.97	44.37–68.60	

Statistically significant values are marked in bold

**Fig. 4:**
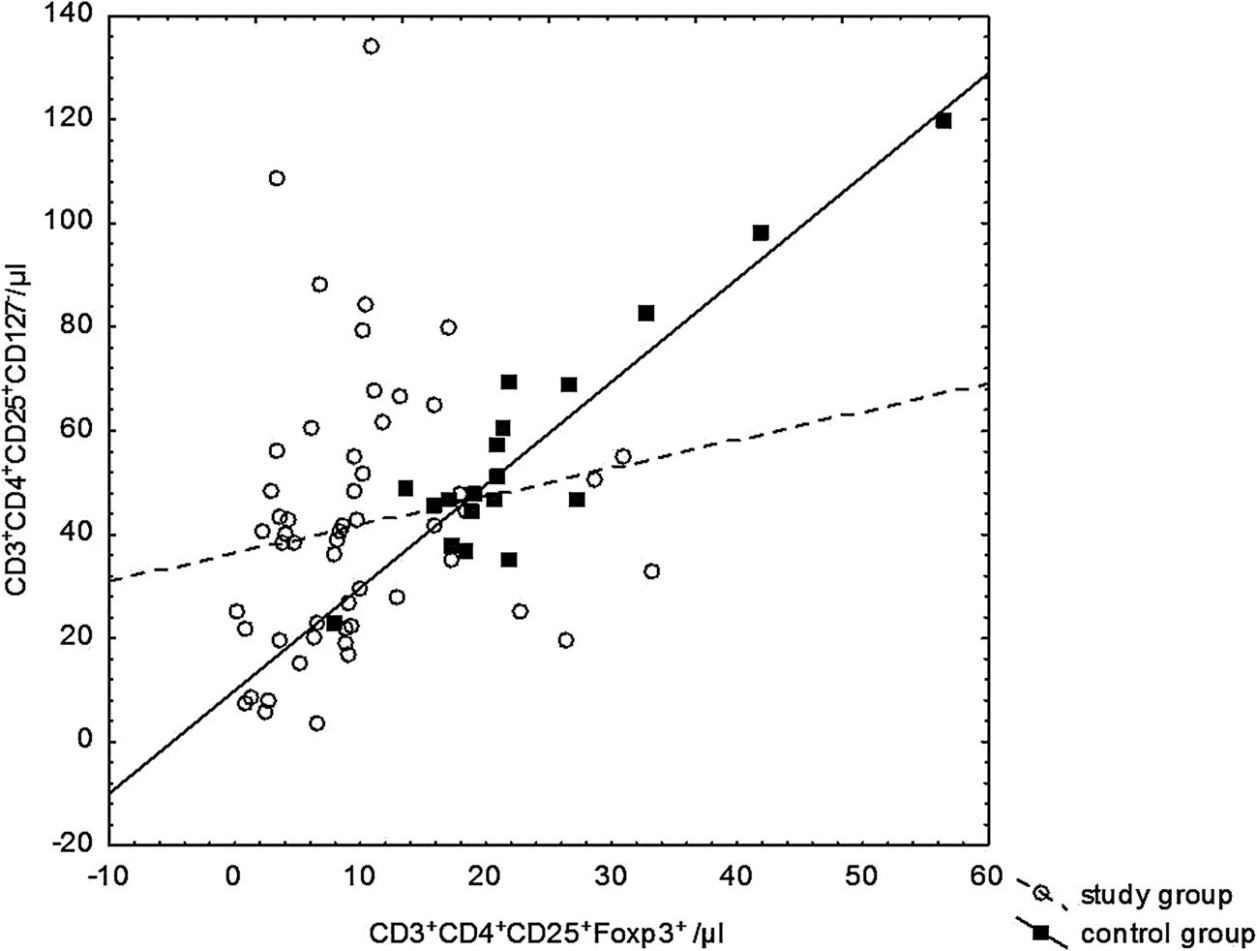
Correlations between regulatory CD3^+^CD4^+^CD25^+^CD127^−^ and CD3^+^CD4^+^CD25^+^Foxp3^+^ cell numbers in the study group and the control group

Statistically significant positive correlations between the absolute number of CD4^+^CD25^+^CD127^−^, and CD4^+^CD25^+^Foxp3^+^ regulatory cells have been observed. The correlation coefficient for the study group was 0.362 (*p* < 0.008) and for the control group 0.674 (*p* < 0.002) (Fig. 4).

**Fig. 5:**
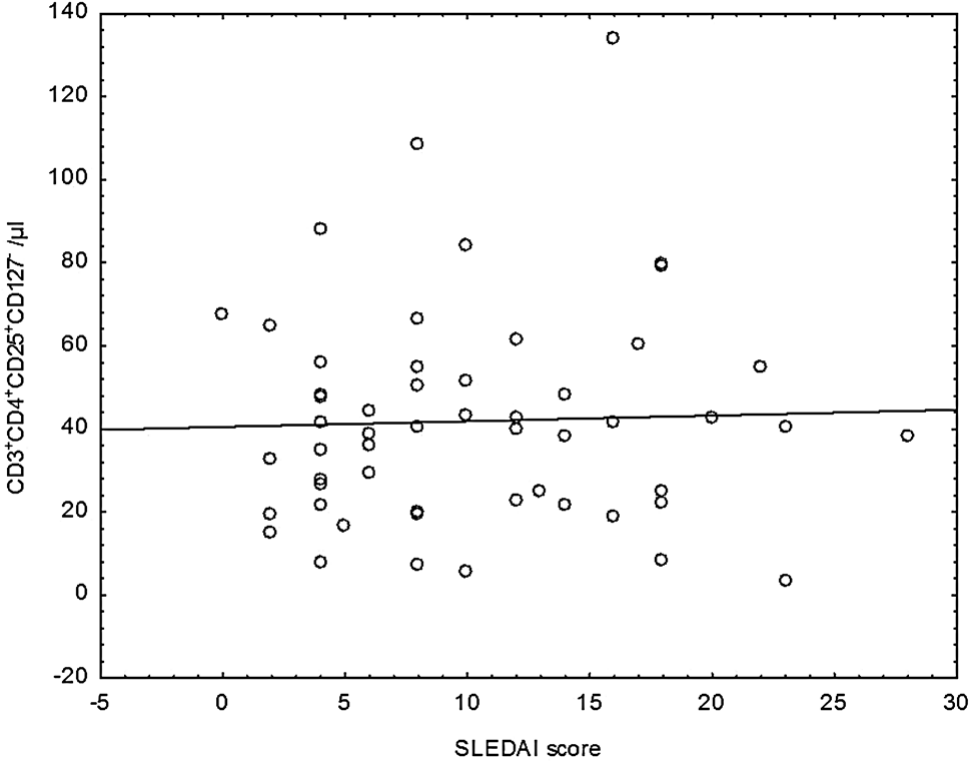
Scatter diagram for the absolute number of CD3^+^CD4^+^CD25^+^CD127^−^ regulatory cells and disease activity index SLEDAI

There were no statistically significant differences between the two patient groups according to disease activity measured by SLEDAI scale, both in percentage and absolute number of CD4^+^CD25^+^CD127^−^ regulatory cells (Fig. 5).

Statistical analysis showed a significantly lower percentage of regulatory CD4^+^CD25^+^CD127^−^ cells in patients with low disease activity (*p* = 0.033) and in patients with high disease activity (*p* < 0.001) compared to the control group. The absolute values of CD4^+^CD25^+^CD127^−^ among groups also demonstrated statistically significant differences. Low disease activity group weakly, but substantially, differed from the control group (*p* = 0.048), also a group with high disease activity differed from the control group (*p* = 0.023). In both cases, in the control group higher absolute values of the regulatory CD4^+^CD25^+^CD127^−^ cells compared to the SLE groups were observed.

The percentages and absolute count of regulatory CD4^+^CD25^+^CD127^−^ cells did not differ significantly between the groups according to rSLEDAI. Significant differences of regulatory lymphocytes between the group with low disease activity measured by the rSLEDAI scale and the control group (*p* = 0.001) have been demonstrated, and the percentage was lower in SLE patients. There were also significantly lower percentages of CD4^+^CD25^+^CD127^−^ regulatory cells in the group with active disease compared to the control group (*p* < 0.001). Lower absolute amounts of the regulatory cells in patients with low disease activity as compared to healthy subjects (*p* = 0.017) and in patients with high disease activity in comparison to the control group (*p* = 0.037) have been shown.

## Discussion

The subpopulation of Treg cells controls the immune response in SLE and other autoimmune diseases (Miyake et al. 2011). The most important marker of Treg cells is though a transcription factor Foxp3, considered essential for the development and activity of this subpopulation (Fontenot et al. 2003; Hori et al. 2003; Zheng and Rudensky 2007). In the present study, the number of Treg cells by the CD4^+^CD25^+^Foxp3^+^ and CD4^+^CD25^+^CD127^−^ phenotypes was determined.

Most previous studies have demonstrated that SLE patients have decreased numbers of Tregs in their peripheral blood than healthy controls (Cai et al. 2012; Habibagahi et al. 2011; Henriques et al. 2010; Ma et al. 2013; Xing et al. 2012). Our analysis has shown that both the percentage and absolute number of CD4^+^CD25^+^Foxp3^+^ regulatory cells are significantly lower in patients with SLE compared to the control group. Values of *p* were the lowest in patients with active disease when compared with the control group, which confirms the pathogenic role of this population in SLE.

In the present study, which is in line with the other authors’ findings (Cai et al. 2012; Ma et al. 2013; Xing et al. 2012), the absolute number of CD4^+^CD25^+^Foxp3^+^ regulatory cells negatively correlated with disease activity measured by the SLEDAI scale, although no significant differences were observed in the number and percentage of these cells derived from patients with inactive (SLEDAI ≤5) and active (SLEDAI >5) phase of disease. What is very important is that the absolute number and the percentage of CD4^+^CD25^+^Foxp3^+^ regulatory lymphocytes were significantly lower in the group with renal involvement compared to the group without renal involvement.

The dependence of the variability of CD4^+^CD25^+^Foxp3^+^ regulatory cell number with SLEDAI and rSLEDAI indices seems to be a promising exponent of disease activity, particularly renal involvement.

We found a statistically lower percentage and absolute number of CD4^+^CD25^+^CD127^−^ regulatory cells in the patient group, compared with the control group. This observation is in line with other literature reports (Henriques et al. 2010; Yang et al. 2009). These relationships, however, had less statistical power compared to those determined using the expression of intracellular transcription factor Foxp3.

Unlike in the case of CD4^+^CD25^+^Foxp3^+^ lymphocytes, no significant differences were observed in the number of CD4^+^CD25^+^CD127^−^ lymphocytes and disease activity measured by the SLEDAI and rSLEDAI scales.

The number of regulatory cells determined by intracellular Foxp3 expression in the present study correlated with regulatory cell number determined by extracellular staining as a CD4^+^CD25^+^CD127^−^ subpopulation. This confirms the correlation of simultaneously exhibiting the high expression of the CD25 molecule and low expression of CD127 molecule with intracellular factor Foxp3 expression (Cai et al. 2012; Crispin et al. 2003). This correlation was much stronger in the control group compared to patients with SLE. The literature data suggest that the expression of the CD127 molecule significantly decreases after the T cells activation, therefore the phenotype of CD4^+^CD25^+^CD127^−^ does not coincide strictly with the expression of Foxp3 and does not correspond to the regulatory phenotype, especially in patients with autoimmune diseases (Aerts et al. 2008).

There is a publication in which the number of Tregs cells was determined using both of these phenotypes in patients infected with human immunodeficiency virus (HIV). Patients were divided into two groups, depending on the viremia. As expected, a strong correlation was found between CD4^+^CD25^+^CD127^−^ and CD4^+^CD25^+^Foxp3^+^ cells in the group without viremia. There was no such correlation in patients with HIV viremia. The results confirm that CD4^+^CD25^+^CD127^−^ phenotype corresponds rather to an activated than regulatory T lymphocytes in the group with HIV viremia, and expression of the CD127 molecule is associated with T cell activation (Del Pozo-Balado et al. 2010).

It has also been shown that, in blood samples from normal healthy donors and patients with systemic scleroderma, about 35 % of CD127^low/−^ cells that did not express Foxp3 and, conversely, about 30 % of CD127^+^ cells expressed Foxp3. It suggests that these markers did not represent the same population of Tregs. The authors suggests that peripheral CD4^+^CD25^+^ Tregs cannot be accurately identified and purified using the surface expression of CD127 as an alternative to the transcription factor Foxp3 (Klein et al. 2010). Expression of CD127 is also downregulated early in the course of activation of Teff cells (Aerts et al. 2008). Furthermore, low expression of CD127 molecule is not an inherent attribute of Tregs, since it is known that Tregs are able to respond to IL-7 (Mazzucchelli et al. 2008).

To summarize, the low surface expression of CD127 in combination with the expression of CD25 does not fully reflect the number of regulatory cells expressing the transcription factor Foxp3. This suggests that the scientific reports related to the variability of CD4^+^CD25^+^CD127^−^ regulatory subpopulation should be interpreted with caution, especially in the context of autoimmune diseases (Klein et al. 2010).

Isolation of pure Treg population is a challenge; thus, differences in the results of the various groups in clinical studies. It is difficult to compare the results directly, both in terms of number and function of Tregs, especially in the inflammatory conditions in clinical samples where there is a considerable amount of activated effector cells. In our opinion, there is a need for more specific markers which will select Treg cells both for diagnostic and therapeutic applications in the strategy based on flow cytometry.

Lack of immunosuppressive therapy impact assessment on determined subpopulations is one of the limitations of the study. Such analysis was not possible due to the small size of the study group. It seems, however, that the possible impact of immunosuppressive therapy on the results was at least partially eliminated by comparing groups of different disease activity proven by well known indicators. This research was not designed as a prospective cohort study. Its aim was to evaluate the number of Tregs subpopulation at one point in groups of different disease activity, and thus the lack of follow-up can be considered a limitation of this study.

In conclusion, our data confirm the reduction in percentage and absolute count of regulatory cells, along with an increase in disease activity. This indicates the importance of Tregs in the inflammatory process and suggests that the reduction in the number of regulatory cells is associated with the development and exacerbation of the disease. The results of the presented study are a part of a discussion on the significance of regulatory cells in the pathogenesis of SLE, highlighting aspects of the immune imbalances and deficits in peripheral tolerance.

## Abbreviations

Anti-dsDNA: Anti-double stranded DNA antibodies
CD: Cluster of differentiation
Foxp3: Transcription factor forkhead box P3
HIV: Human immunodeficiency virus
LN: Lupus nephritis
mRNA: Messenger RNA
rSLEDAI: Renal systemic lupus erythematosus disease activity index
SLE: Systemic lupus erythematosus
SLEDAI: Systemic lupus erythematosus disease activity index
Teff: Effector T lymphocyte
Treg: Regulatory T lymphocyte
PBS: Phosphate-buffered saline
FBS: Fetal bovine serum
